# Using Fused Data from Perimetry and Optical Coherence Tomography to Improve the Detection of Visual Field Progression in Glaucoma

**DOI:** 10.3390/bioengineering11030250

**Published:** 2024-03-03

**Authors:** Leo Yan Li-Han, Moshe Eizenman, Runjie Bill Shi, Yvonne M. Buys, Graham E. Trope, Willy Wong

**Affiliations:** 1 The Edward S. Rogers Sr. Department of Electrical & Computer Engineering, University of Toronto, Toronto, ON M5S 3G4, Canada; 2 Department of Ophthalmology & Vision Sciences, University of Toronto, Toronto, ON M5T 3A9, Canada; 3 Temerty Faculty of Medicine, University of Toronto, Toronto, ON M5S 1A8, Canada; 4 Institute of Biomedical Engineering, University of Toronto, Toronto, ON M5S 3E2, Canada

**Keywords:** autoencoder, data fusion, glaucoma progression, optical coherence tomography, perimetry, visual field

## Abstract

Perimetry and optical coherence tomography (OCT) are both used to monitor glaucoma progression. However, combining these modalities can be a challenge due to differences in data types. To overcome this, we have developed an autoencoder data fusion (AEDF) model to learn compact encoding (AE-fused data) from both perimetry and OCT. The AEDF model, optimized specifically for visual field (VF) progression detection, incorporates an encoding loss to ensure the interpretation of the AE-fused data is similar to VF data while capturing key features from OCT measurements. For model training and evaluation, our study included 2504 longitudinal VF and OCT tests from 140 glaucoma patients. VF progression was determined from linear regression slopes of longitudinal mean deviations. Progression detection with AE-fused data was compared to VF-only data (standard clinical method) as well as data from a Bayesian linear regression (BLR) model. In the initial 2-year follow-up period, AE-fused data achieved a detection F1 score of 0.60 (95% CI: 0.57 to 0.62), significantly outperforming (*p* < 0.001) the clinical method (0.45, 95% CI: 0.43 to 0.47) and the BLR model (0.48, 95% CI: 0.45 to 0.51). The capacity of the AEDF model to generate clinically interpretable fused data that improves VF progression detection makes it a promising data integration tool in glaucoma management.

## 1. Introduction

Glaucoma is a progressive optic neuropathy characterized by irreversible vision loss and abnormal thinning of the retinal nerve fiber layer (RNFL) [1]. Visual field (VF) testing, also known as perimetry, is the primary clinical test for assessing functional vision loss in glaucoma, while optical coherence tomography (OCT) is the standard imaging tool for evaluating the structural integrity of the retinal nerve fiber layer. The subjective and probabilistic nature of VF testing introduces significant variability and can lead to delays in detecting disease progression [2]. While OCT data, such as peripapillary RNFL thickness, is less susceptible to subjective errors, recent research underscores the inadequacy of relying solely on a single modality for glaucoma monitoring [3,4,5]. This is due to the fact that at different stages of the disease, glaucoma progression is associated with changes in either VF or OCT measurements and these changes can be asynchronous [3,4,5].

Consequently, there is a pressing need to integrate information from VF and OCT measurements to accurately detect glaucoma progression. However, integration poses challenges owing to substantial differences in scales, dimensions, and variability between data from the two modalities. Clinical perimeters, like the Humphrey Field Analyzer (Carl Zeiss Meditec, Inc.; Dublin, CA, USA), provide combined functional and structural reports yet manual interpretation can be subjective and relies heavily on clinical experience. One potential solution is to use machine-learning techniques as these offer a data-driven approach without the need to assume fixed relationships between the different data streams. However, the black-box nature of machine-learning algorithms presents a challenge in terms of interpretability and explainability, making it difficult for clinicians to trust the results generated from such algorithms. 

With respect to data fusion studies in glaucoma, a number of methods have been developed to integrate differential light sensitivity (DLS) measurements from VF testing and RNFL thickness data from OCT. Bizios et al. [6] developed a model to fuse VF and OCT data based on manually defined rules that consider the spatial correspondence between functional and structural measurements. The fused data was then used to train an artificial neural network (ANN) for glaucoma diagnosis. While detecting glaucoma cases with the fused data outperformed models trained with VF data alone (i.e., using pattern deviation maps), the complexity of the fused data limits clinical interpretability of the results. Similarly, in a recent study, Song et al. [7] developed a deep-learning ANN model to integrate global and regional features of VF and OCT measurements. The authors showed that the accuracy of diagnosing glaucoma using the proposed model surpassed that of single-modal approaches. However, the difficulty in tracing through the decision-making process of a deep-learning model can pose challenges in regard to the comprehension of the diagnostic results. Wu and Medeiros [8] proposed a structure-function index to combine information from VF and OCT measurements for glaucoma diagnosis. This method first transforms data from the two testing modalities to the same scale. Subsequently, the transformed data is combined based on predefined rules taking into consideration the difference in measurement ranges of the two data types across varying degrees of glaucoma severity. Although the combined structure-function index exhibited superior diagnostic performance compared to using data from a single modality alone [8], the reliance on hard-coded rules for the data transformation and integration may not account for the variability observed during disease progression. Furthermore, the combined index is challenging to interpret in terms of the established clinical criteria for glaucoma diagnosis.

The above methods are aimed primarily at improving glaucoma diagnosis, and not for the detection of VF progression. A tool for monitoring glaucoma progression should not only indicate whether an eye is deteriorating but also have the capacity to measure the rate of progression [9,10,11]. Medeiros et al. [11] developed a Bayesian hierarchical linear model that combines changes in visual field index measurements from perimetry and average RNFL thickness data from scanning laser polarimetry. In this method, statistical parameters that describe rates of change in a cohort of glaucoma patients were used as the a priori information to estimate subject-specific progression rates. While the method demonstrated improved performance in detecting progression, the complexity of the model poses challenges in its interpretability. Russell et al. [12] developed a Bayesian linear regression (BLR) model that uses changes in the neuroretinal rim area to improve estimates of VF progression rates. Results from the BLR model are easily interpretable, and the combined progression rates demonstrated reduced error in predicting future VF measurements, which can be further used for progression analysis. However, there are limitations to this approach as the relationship between functional and structural measurements is likely nonlinear.

Inspired by these earlier studies, the objective of this paper is to develop a method that fuses structural RNFL thickness data and functional VF measurements to improve the detection of VF progression. To overcome the nonlinear and changing relationship between structural and functional deteriorations in different glaucoma stages, we employed a trainable ANN model rather than a method based on fixed rules. An ANN is well-suited for describing complex and nonlinear relationships between data sets with substantial differences in scales, dimensions, and variability. Moreover, as fused data should be interpreted in a similar manner to that of data from a standard clinical VF test, we used an autoencoder (AE) to construct the data fusion model. The AE model facilitates a compact representation of input data, referred to as an encoding. This encoding retains essential features required to reconstruct the original input data while removing any redundancies [13], thus making it a widely used technique for multimodality data fusion across diverse fields [14,15,16,17]. In what we believe is a key and significant contribution to this area, we introduce an “encoding loss function” to regularize the fused data so that the encoding has a similar structure to that of the VF test. The fused data can then be interpreted in the same way that a visual field test is interpreted, taking a crucial step towards enhancing the clinical interpretability of the machine-learning model’s results, thereby addressing a key drawback of conventional AE data fusion models. Finally, to evaluate the efficacy of the AE data fusion (AEDF) model in glaucoma monitoring, we compared VF progression detection performance using only VF data (the standard clinical method), data generated by the BLR model, and data generated by the AEDF model. 

## 2. Materials and Methods:

### 2.1. VF and OCT Data

**Figure 1:**
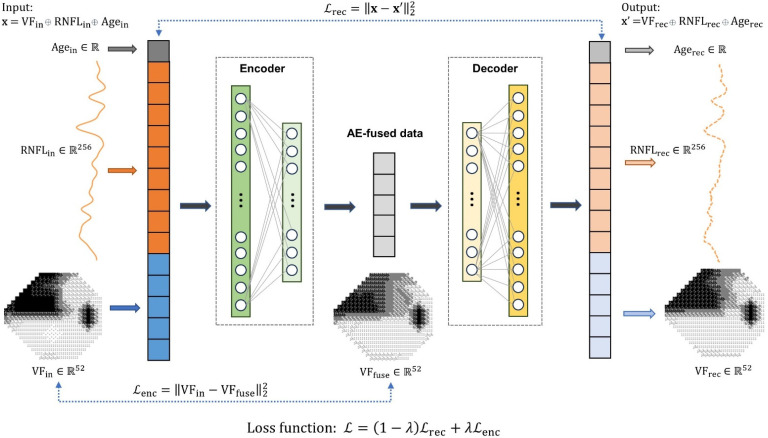
The overall architecture of the autoencoder (AE) data fusion model. The input to the model is a vector that includes pointwise differential light sensitivity thresholds from visual field (VF) testing (52-dimensional vector), retinal nerve fiber layer (RNFL) thickness profile (256-dimensional vector), and patient’s age at the time of the test (scalar). The encoder network, constructed with a two-hidden layer multilayer perceptron (MLP) model, processes the input vector and generates a 52-dimensional encoding vector as the AE-fused data. The decoder network, a symmetrically structured MLP model, aims to reconstruct the input data from the encoding vector. The reconstruction loss ($L_{\mathrm{rec}}$) is the mean squared error (MSE) between the input and output vectors of the AE data fusion model. The encoding loss ($L_{\mathrm{enc}}$) is the MSE between the AE-fused data and the measured VF. The training objective is to minimize the convex combination of the reconstruction loss and the encoding loss, weighted by a scalar λ.

**Figure 2:**
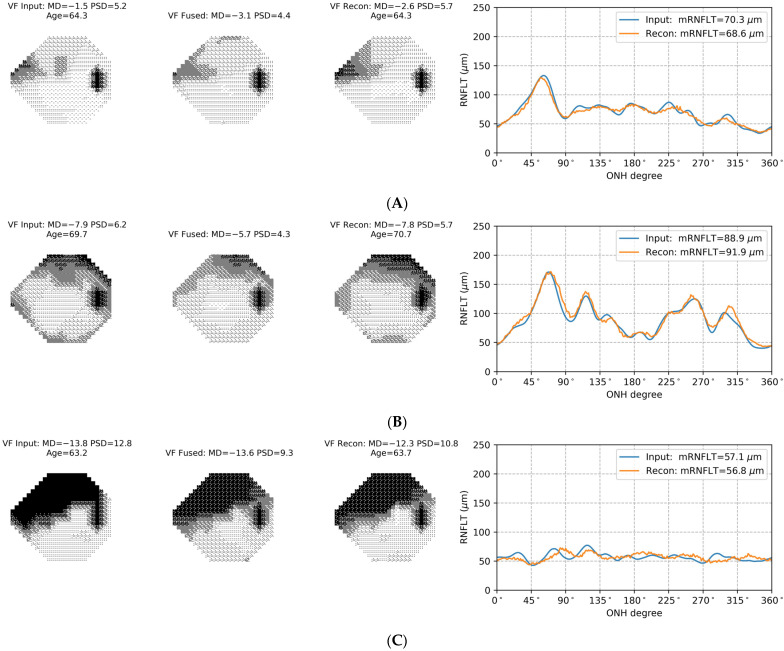
Examples of the autoencoder (AE) data fusion model for eyes with mild (panel **A**), moderate (panel **B**), and severe (panel **C**) VF defects. In each panel, the three visual field (VF) plots represent the input VF to the AE data fusion model (left), the AE-fused data (middle), and the reconstructed VF (right). The right graph in each panel illustrates the input retinal nerve fiber layer thickness (RNFLT) profile data to the AE data fusion model (blue curve) and the reconstructed RNFLT profile data (orange curve) from the AE data fusion model. The RNFLT profile data, a 256-dimensional vector, is visualized as a curve of the RNFLT, where the horizontal axis represents the angular position (0 to 360 degrees) around the optic nerve head (ONH), and the vertical axis represents the RNFL thickness measurement (in μm). These examples provide visualized representations of the way that the AE data fusion model dynamically combines results from VF and OCT tests.

In this retrospective study, we evaluated 2504 pairs of longitudinal VF and OCT tests of 140 glaucoma patients who had been followed for a minimum of four years at the glaucoma clinic of the Toronto Western Hospital. Each pair of VF and OCT tests was performed on the same day. All VF tests were conducted using the Humphrey Field Analyzer with the 24-2 SITA Standard algorithm. Each VF consists of 54 differential light sensitivity thresholds at discrete locations extending 30 degrees around the central fixation point. The two VF testing points that are mapped onto the optic nerve head (ONH), i.e., the blind spot, were not used in this study. The ONH, where retinal ganglion cell axons exit the retina and converge to form the optic nerve, represents the primary site of structural damage due to glaucoma. The peripapillary RNFL thickness profile data obtained from OCT encompasses 256 A-scans along a 3.45 mm circle centered at the ONH. This data is typically recorded as a 256-dimensional vector, with each element representing a thickness measurement of the RNFL (in μm) at a particular angular position (0 to 360 degrees) around the ONH (see Figure 1 and Figure 2). All RNFL thickness profile data utilized in this study was acquired using the Cirrus HD-OCT (Carl Zeiss Meditec, Inc.; Dublin, CA, USA). Only pairs of reliable VF and OCT tests were analyzed. In line with prior research [8], the reliability criteria for VF data were false-positive and false-negative rates < 15% and fixation loss rate < 33%. For OCT data, the reliability criterion was defined as signal strength ≥ 7. Patients with severe VF defects, i.e., mean deviation (MD) worse than −20 dB on the first test (visit), were excluded from model training and evaluation, as both VF and OCT changes are notably affected by the floor effect in measurements of advanced glaucoma [18]. Additionally, OCT tests containing missing or corrupted RNFL thickness data points were also excluded. 

The study received approval from the Research Ethics Board of the University Health Network, Toronto, Ontario, Canada, and adhered to the tenets of the Declaration of Helsinki.

### 2.2. Data Fusion Models for Function-Structure Measurements

#### 2.2.1. Autoencoder Data Fusion Model

The AE data fusion (AEDF) model consists of two components: (1) an encoder network ${z=f}_{\theta }\left(x\right)$ parameterized by θ, which maps the input data $x\in R^{d_{x}}$ to a low dimensional encoding space $z\in R^{d_{z}}$ ($d_{x}>d_{z}$), where the encoder’s output is referred to as the AE-fused data, and (2) a decoder network $x^{\prime }=g_{\phi }\left(z\right)$ parameterized by ϕ, aiming to reconstruct the input data from the encoding space, such that $x^{\prime }\in R^{d_{x}}$ [13]. Here, $d_{x}$ and $d_{z}$ represent the dimensions of the input and encoding spaces, respectively.

Given that similar defects in visual field or retinal structure at different ages may lead to different clinical interpretations, we used the patient’s age at the time of testing, a (where a∈R), as one of the input parameters to the AEDF model. The other input parameters are the VF pointwise DLS thresholds, denoted as v (where $v\in R^{52}$), and the RNFL thickness profile, denoted as r (where $r\in R^{256}$). As such, the dimension of the input to the AE data fusion model, denoted as x=(v, r, a), is $d_{x}=309$. Note that in Section 3.5, we described sensitivity analysis that investigates the contribution of the input parameters to the detection of VF progression. 

In a similar manner, the output of the decoder is expressed as x′=(v′, r′, a′), where $v^{\prime }\in R^{52},\;r^{\prime }\in R^{256}$, and $a^{\prime }\in R$ represent the reconstructed VF, RNFL thickness profile, and patient’s age, respectively. Furthermore, the encoding space has the same dimensionality as the input VF data, i.e., $d_{z}=52$ for the 24-2 pattern, so that the AE-fused data (z) can be interpreted in the same space as the data from the VF test.

In the training phase, parameters of the encoder and decoder were simultaneously updated to minimize the reconstruction loss $L_{rec}$, which was defined as the mean squared error (MSE) between the input and reconstructed data (Equation (1)):

(1) $$L_{rec}\left(g_{\phi }\left(f_{\theta }\left(x\right)\right),\;x\right)=\frac{1}{N}\sum_{i=1}^{N}{\left(x_{i}^{\prime }-x_{i}\right)}^{2}\;\;\;$$

Here, $x_{i}$ and $x_{i}^{\prime }$ represent the input and reconstructed vectors of the i-th sample, respectively, and N is the total number of training examples. Training the AEDF model by solely minimizing the reconstruction loss, as defined in Equation (1), does not ensure that the AE-fused data (the output of the encoder) will retain the appearance of standard VF tests. 

To address this challenge, we introduced an encoding loss, in addition to the reconstruction loss, to the model training. Specifically, the encoding loss function was defined as the MSE between the encoding and the input VF data, formulated as $L_{enc}=\frac{1}{N}\sum_{i}^{N}{\left(z_{i}-v_{i}\right)}^{2}$, where $z_{i}$ and $v_{i}$ are the encoding and measured VF data of the i-th sample, respectively. With the incorporation of the encoding loss, each point in the AE-fused data represents a modified version of the differential light sensitivity from the input VF, making it interpretable by standard clinical methods for VF progression analysis. Thus, it is unnecessary to develop new criteria for the AE-fused data to detect progression, as it aligns with existing standard clinical techniques to analyze VF data.

To this point, the total loss function (L) for the training of the AEDF model was defined as the convex combination of the reconstruction loss and the encoding loss, formulated as:

(2) $$L={\left(1-\lambda \right)L}_{rec}+{\lambda L}_{enc}=\frac{1-\lambda }{N}\sum_{i=1}^{N}{\left(x_{i}^{\prime }-x_{i}\right)}^{2}+\frac{\lambda }{N}\sum_{i=1}^{N}{\left(z_{i}-v_{i}\right)}^{2}$$

where λ∈[0, 1] is a hyperparameter that controls the strength of regularization from the encoding loss. As suggested in Equation (2), the reconstruction and encoding losses work in an adversarial manner, meaning a reduction in one loss results in an increase in the other. In our experiments, a grid search was performed to determine the λ that leads to the best performance in the detection of VF progression (optimal λ). The effects of introducing encoding loss and the selection of λ on the AE-fused data are discussed later.

Figure 1 illustrates the overall architecture of the proposed AEDF model. The encoder and decoder networks were constructed using symmetrical structures of multilayer perceptron models with two hidden layers. Gaussian error linear unit (GELU) activation function [19] and layer normalization technique [20] were adopted to accelerate convergence in model training. The performance of the AE data fusion model was assessed using 10-fold cross-validation (CV). In each CV fold, the data from the same eye was not used simultaneously in both training and validation. Meanwhile, the data distribution in terms of disease severity was held constant across all training and validation sets. All data were normalized to the range between 0 and 1, based on their corresponding minimum and maximum values, and were converted back to the original scales for evaluation and visualization. The AE data fusion model was implemented using the deep learning platform PyTorch v1.13.0 [21] for Python. 

#### 2.2.2. Bayesian Linear Regression Model

To serve as a baseline comparison for the AE data fusion model, we implemented a Bayesian linear regression (BLR) model that combines the progression rates of structural and functional measurements. This model is based on the work of Russell et al. [12], who used the linear regression slope of the neuroretinal rim area as a priori information for the posterior progression rate of VF mean-sensitivity (MS). Since our study involved a different type of structural data, we utilized the mean RNFL thickness data from OCT measurements to derive the prior distribution of the progression rate. Additionally, we measured the MD slope rather than the MS slope to maintain consistency with clinical methods to detect VF progression. These modifications do not introduce significant changes to Russell et al.’s work [12], as the MD and MS slopes in our data are comparable in value (−0.21 ± 0.44 dB/year for MD slope vs. −0.26 ± 0.42 dB/year for MS slope). Moreover, the RNFL thickness data used to derive the prior was also suggested as a possible extension in their original study [12]. 

In general, the posterior progression rate for the BLR model is a weighted average of the likelihood (functional) and prior (structural) progression rates, with the weights determined by the variances of the functional and structural measurements so that data with lower variability receives a higher weight in determining the posterior progression rate. More details on the BLR model can be found in the Appendix A.

### 2.3. Performance Evaluation

For performance evaluation, we first implemented a data augmentation strategy by dividing each longitudinal VF series for each eye (both measured VF and AE-fused data) into short-term segments using variable-length sliding windows. In this way, long-term gradual VF progression was represented by multiple short-term progressions with different progression rates, thereby enhancing data diversity. Moreover, this segmentation strategy helped mitigate the influence of nonlinearity in long-term measurements, making the clinical linear model a better fit for VF progression detection. Note that the nonlinear trend is commonly associated with the physiological nature of glaucoma and interventions in management [22,23]. As a result, the evaluation metrics obtained with the segmented data are likely to be more representative of the real performance in detecting progression. 

A sliding window of 4, 5, 6, 7, and 8 years was applied to both the longitudinal AE-fused data and the measured VF data for each eye to generate increasingly longer data segments. These segments of measured VF data were used to determine the ground truth label of progression via the calculation of a linear regression slope of MD. The segments of AE-fused data were assigned the same ground truth labels as their corresponding measured VF segments. The criterion for VF progression was defined as the MD deteriorating at a rate worse than 0.5 dB per year (i.e., MD linear regression slope <−0.5 dB/year), a common clinical indicator for moderate VF progression [24]. Classification of VF progression was then conducted based on segments of AE-fused and measured VF data from the 1–3 years of data in each segment (in 0.5-year intervals). The classification results were compared to the corresponding ground truth labels to derive the sensitivity, specificity, and F1 score in classifying VF progression. The F1 score is an evaluation metric that uses a single numerical measure to describe the classifier’s capacity to correctly identify true positives and avoid false positives, providing a comprehensive assessment of the performance. The evaluation metrics are defined as follows:

$$Sensitivity=\frac{TP}{TP+FN}$$

$$Specificity=\frac{TN}{TN+FP}$$

$$F1=\frac{2TP}{2TP+FP+FN}$$

where TP, TN, FP, and FN represent true positives, true negatives, false positives, and false negatives in the classification, respectively. 

Furthermore, we aggregated metrics obtained with different sliding windows at each time point, forming the confidence interval for classification performance with longitudinal VF data of various durations. These aggregated metrics for AE-fused data segments were compared to those from measured VF segments and to results from the BLR model to assess the model’s effectiveness in detecting VF progression. In these comparisons, the Wilcoxon signed-rank tests were used to determine statistical significance. Statistical analyses were performed with the SciPy library [25] for Python.

## 3. Results

### 3.1. Data Characteristics

A total of 2504 pairs of reliable VF and OCT tests from 253 eyes of 140 glaucoma patients were included in this study. Across all patients, the average age at the first visit was 63.7 ± 11.8 years (mean ± standard deviation), ranging from 29.7 to 88.5 years. The average follow-up length was 7.7 ± 1.7 years (range: 4.2 to 10.6 years), with a mean number of visits of 9.9 ± 3.7. For all eyes, the average mean deviation (MD) in the first VF test was −3.2 ± 5.8 dB, with a mean progression rate (linear regression slope) of −0.21 ± 0.44 dB/year. The mean RNLF thickness for the first OCT test was 78.7 ± 14.4 μm, with an average progression rate of −0.24 ± 0.97 μm/year. Detailed data characteristics of the cohort are summarized in Table 1. 

### 3.2. Autoencoder Data Fusion Model 

We first investigated the reconstruction performance of the AE data fusion (AEDF) model, as it is a crucial factor in determining the model’s ability to represent information from both VF and OCT tests. Over the 10-fold cross-validation, the AEDF model achieved an average pointwise mean absolute error (MAE) of 2.0 dB for VF reconstruction in the testing phase, with a 95% confidence interval (CI) ranging from 1.8 to 2.4 dB. For RNFL thickness data, the AEDF model had an average reconstruction MAE of 3.6 μm (95% CI: 2.8 to 4.4 μm) in testing. Additionally, the average pointwise MAE between the input VF data and AE-fused data (representing the encoding loss) was 2.5 dB (95% CI: 2.1 to 2.9 dB). This high-level reconstruction performance demonstrated the model’s effectiveness in extracting and integrating representative features from both modalities into the resulting fused data. Meanwhile, the relatively low encoding loss indicated that the AE-fused data maintained good consistency with VF measurements, which assures its clinical interpretability. This will be explained next in more detail. 

Figure 2 provides examples of data from the AEDF model for eyes with mild (Figure 2A), moderate (Figure 2B), and severe (Figure 2C) VF defects. Each example shows the input data, AE-fused data, and the output reconstructed data, providing visualized representations of the way that the AE data fusion model combines results from the two testing modalities. For the eye with mild VF defect (Figure 2A), the RNFL thickness measurements (the rightmost plot) exhibit notable thinning in the 225° to 315° region. As such, the mean RNFL thickness (70.3 µm) falls below the normal range of 75.0 µm to 107.2 µm suggested by the Cirrus HD-OCT device [26]. This localized RNFL thinning is reflected in the AE-fused data as a VF defect of more depression in the superior nasal region of the field (the middle VF plot). It should be noted that the region where the VF loss lies in the AE-fused data matches the area of RNFL thinning according to the structure-function map [27]. Since the VF and OCT data describe the same defect, the AE-fused data tend to have a lower MD (−3.1 dB in the middle VF plot) than the MD of measured VF data (−1.5 dB in the left VF plot). 

In Figure 2B, the measured VF shows a moderate defect with MD of −7.9 dB. In this field, the defect pattern is a superposition of an actual VF loss in the superior field and lens rim artifact. Considering that the RNFL thickness in this eye is overall normal (mRNLFT = 88.9 µm), the impact of lens rim artifacts is removed in the AE-fused data (the middle VF plot), leading to milder VF loss (MD = −5.7 dB), while maintaining the arcuate defect pattern in the superior field. Note that the reconstructed VF data (the right VF plot) maintains good consistency with the measured VF data in terms of the shape and the depth of the defect (MD = −7.8 dB), showing that the information from the measured VF test has been embedded into the AE-fused data. 

In the advanced glaucoma case shown in Figure 2C, the floor effect dominates the RNFL thickness measurements, plateauing at the level of around 50 µm (the rightmost plot). In this case, the resulting AE-fused data (the middle VF plot) is more dependent on data from VF testing and, correspondingly, shows greater consistency (MD = −13.6 dB) with the measured VF data (MD = −13.8 dB).

### 3.3. Detecting VF Progression 

As discussed in the Section 2, each long-term VF and OCT data series was divided into multiple short-term segments to increase the number of cases with progressing and stable VF series. Using the segmented data, we compared the performance of detecting VF progression with AE-fused data, measured VF data (clinical data), and data from the BLR model. 

For all three methods, we computed specificity, sensitivity, and F1 scores for VF progression using data collected over the first two years of the follow-up period in each segment. The selection of a two-year period was based on the minimum suggested follow-up duration for reliable estimation of VF progression rate [24]. In the initial 2 year follow-up, the specificity of detecting VF progression using AE-fused data was 0.70 (95% CI: 0.68 to 0.71), representing a 94% improvement (*p* < 0.001) over the detection specificity with the measured VF data (0.36, 95% CI: 0.35 to 0.38) and a 27% improvement (*p* < 0.001) over the detection specificity when using data from the BLR model (0.55, 95% CI: 0.54 to 0.57). In the same period (i.e., the initial 2 years), the sensitivity of detecting VF progression with the AE-fused data was 0.53 (95% CI: 0.47 to 0.58), outperforming that of the BLR model (0.35, 95% CI: 0.31 to 0.38, *p* < 0.001) and insignificantly lower than that of using measured VF data (0.54, 95% CI: 0.51 to 0.58, *p* = 0.291). When considering both specificity and sensitivity, the F1 score for VF progression detection with AE-fused data was 0.60 (95% CI: 0.57 to 0.62), surpassing the F1 scores obtained with the measured VF data (0.45, 95% CI: 0.43 to 0.47, *p* < 0.001) and data from the BLR model (0.48, 95% CI: 0.45 to 0.51, *p* < 0.001).

**Figure 3:**
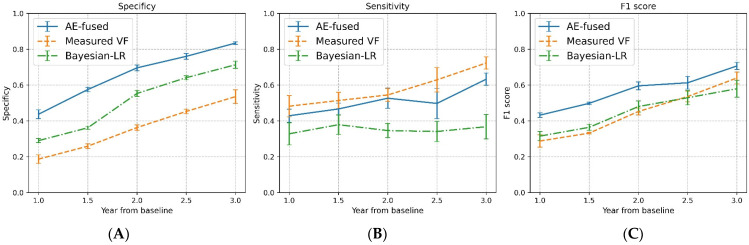
Specificity (panel **A**), sensitivity (panel **B**), and F1 scores (panel **C**) for the detection of visual field (VF) progression using data generated by the autoencoder data fusion model (blue), data of VF measurements (orange), and data from the Bayesian linear regression model (green) at different time points. The x-axis shows the time point, ranging from 1 to 3 years relative to the first test, in which the detection (classification) was made. Each point on the curves is the average performance for the VF time series with various lengths (ranging from 4 to 8 years), with error bars presenting the 95% confidence intervals. As expected, the overall detection performance, measured by F1 scores, for all three data models improved when the number of available data points for the detector increased, i.e., longer time along the x-axis. At different time points, the overall VF progression detection performance (F1 scores) with AE-fused data consistently outperformed the other two methods.

Figure 3 shows the performance of VF progression detection (specificity, sensitivity, and F1 scores) with the three data models at different time points over the initial three years of the follow-up period. As observed, the F1 scores with the AE-fused data consistently outperformed those with only VF data (clinical method) or data from the BLR model. Moreover, the improved performance with AE-fused data was mainly attributed to a significant increase in the detection specificity compared to the other two methods. 

For the results presented above, VF progression was defined as a sequence of VFs in which the MD linear regression slope is worse than −0.5 dB/year. Considering that there is no consensus on the criteria for VF progression, clinics may adopt different thresholds for the detection of VF progression. We investigated the robustness of the detection performance when the criteria for VF progression was either relaxed (MD slope < −0.2 dB/year) or became stricter (MD slope < −1.0 dB/year). 

Table 2 shows a summary of the VF progression detection performance (specificity, sensitivity, and F1 scores) for AE-fused data, measured VF data, and the BLR model’s data in the initial 2 years, for the above three criteria for VF progression. Detection with AE-fused data achieved the highest F1 scores for all three thresholds, demonstrating that the performance gained by using the AE data fusion model is robust to variation in the VF progression criteria. Moreover, the performance patterns for the AE-fused data compared to the other methods were also consistent across different selections of the progression criteria, i.e., substantial improvement in specificity while keeping sensitivity approximately the same.

### 3.4. Selection of λ in the Loss Function

When training the AE data fusion model, the hyperparameter λ was used to balance the contributions of the reconstruction and encoding loss terms. We conducted a grid search for the optimal λ that leads to the best VF progression detection performance by varying λ from 0 to 1, in steps of 0.1. The result showed that when λ=0.6 (optimal λ), the best overall VF progression detection performance can be achieved, with the F1 score of 0.60. 

**Figure 4:**
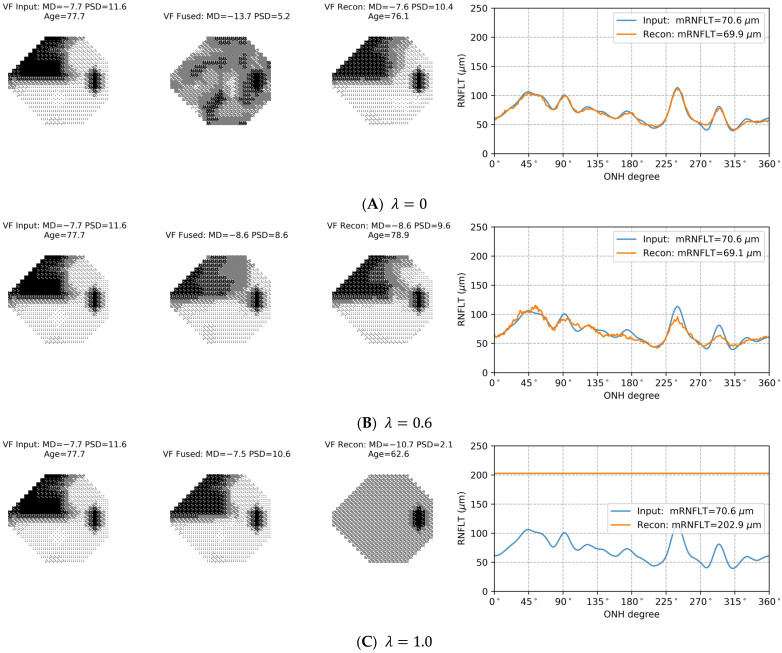
Examples of the autoencoder (AE) data fusion model trained with different λ selections in the loss function. When λ=0 (panel **A**), the training objective of the AE data fusion model was to only minimize reconstruction loss. As such, the AE-fused data (the middle visual field [VF] plot) are so different from the input/measured VF (the left VF plot) that they cannot be interpreted and analyzed with clinical methods. When λ=1 (panel **C**), the training objective was solely to minimize the encoding loss without considering reconstruction loss. The AE-fused data (the middle VF plot) closely resembles the measured VF (the left VF plot) so that it barely contains additional information from retinal nerve fiber layer thickness measurements. With λ=0.6 (panel **B**), both reconstruction and encoding losses contribute to the training of the AE data fusion model. The AE-fused data can be interpreted with clinical knowledge in terms of the VF defect pattern and depth while incorporating sufficient information from both structural and functional tests.

Figure 4 presents three examples that demonstrate the interaction between reconstruction and encoding losses with different λ values. When the training objective of the AE data fusion model was to only minimize reconstruction loss (Figure 4A, λ=0), the VF defect pattern and DLS thresholds of the AE-fused data (the middle VF plot) are so different from the measured VF that the AE-fused data cannot be interpreted in a manner similar to that of the measured VF data. Note that the MD of the fused data in Figure 4A is −13.7 dB, whereas the MD of the measured VF is −7.7 dB. Consequently, standard clinical VF progression detection techniques cannot be used to analyze the AE-fused data when λ=0, even though the AE-fused data retains sufficient information from both testing modalities to reconstruct the input data (low reconstruction errors for both VF and RNFL thickness data). When the training objective is to minimize the encoding loss without considering reconstruction loss (Figure 4C, λ=1), the fused data becomes so akin to the input VF data that it fails to adequately represent information from the RNFL thickness measurements. As a result, the detection performance remains the same as that of using only measured VF data. When both reconstruction and encoding losses contribute to the detection performance (Figure 4B, λ=0.6), the AE-fused data can be interpreted in the same framework as the measured VF data while incorporating sufficient information from structural OCT measurements to improve the detection of VF progression.

### 3.5. Sensitivity to Input Parameters

We carried out a sensitivity analysis to examine the contribution of various input parameters to the performance of VF progression detection. When using both VF and RNFL thickness data as the input to train the AE data fusion model, the detection specificity with the initial two years of AE-fused data significantly outperformed that with measured VF data alone (0.64 vs. 0.36, *p* < 0.001). Meanwhile, the detection sensitivity with the AE-fused data containing both VF and RNFL thickness information showed no substantial difference from that obtained using measured VF data (0.52 vs. 0.54, *p* = 0.178). Moreover, in addition to the VF and RNFL thickness data, when incorporating the patient’s age information into the AE data fusion model, the detection specificity of the AE-fused data can be further improved from 0.64 to 0.70 (*p* < 0.001) while maintaining the sensitivity at the same level (0.53 vs. 0.52, *p* = 0.313).

## 4. Discussion

In this study, we present a method to improve the detection of VF progression by combining differential light sensitivity data from perimetry and RNFL thickness profile data from OCT using an autoencoder data fusion (AEDF) model. Unlike previous methods that rely on statistical or fixed rules for multimodality data integration [6,8,12], the AEDF model learns the function-structure interrelations in glaucoma from patients’ perimetry and OCT data. This data-driven approach offers more flexibility and accuracy in describing nonlinear relationship between structural and functional measurements throughout the course of glaucoma progression. Moreover, a key contribution of our approach is the introduction of an encoding loss function that helps structure the fused data similar to the input VF data, allowing for an easy and intuitive interpretation of the model’s results.

The overall VF progression detection performance (measured by the F1 score) when using the initial two years of AE-fused data was 33% better than the clinical standard method of using only VF data (*p* < 0.001). The improved detection performance was mainly attributed to a significant increase of 94% in detection specificity. When compared with the Bayesian linear regression model, VF progression detection sensitivity and specificity with AE-fused data were enhanced by 51% and 27%, respectively, leading to 25% improvement (*p* < 0.001) in the F1 score. Furthermore, the performance improvement with the AE-fused data is robust to changes in the criteria used to determine VF progression and to the length of the follow-up period.

The loss function employed in the training of the AE data fusion model comprises reconstruction and encoding loss terms, with a weight factor (λ) that controls the relative contributions of the two loss terms. As shown in Figure 4, λ can be used to change the AE-fused data, i.e., the output of the encoder, by adjusting the effect of structural OCT measurements on the VF data. With the optimal λ for VF progression detection (λ=0.6), the appearance of AE-fused data is similar to that of the measured VF data so that the AE-fused data can be interpreted and analyzed by standard methods that are used with VF measurements. At the same time, the combination of reconstruction and encoding losses through λ assures that the AE-fused data incorporate representative features associated with structural measurements from OCT (see Figure 2), which contributes to improved VF progression detection. 

The sensitivity analysis in the Section 3 showed that including the patient’s age in the input of the AEDF model played a role in enhancing detection of VF progression. This observation aligns with data showing that aging is a major risk factor for glaucoma progression [28]. For that reason, it can be expected that VF progression detection could be further improved by incorporating other parameters that are associated with glaucoma into the AEDF model. Such inputs may include intraocular pressure, cup-to-disc ratio, fundus images, or macular OCT measurements, etc. This extended multimodality data integration can be easily realized by expanding the input to the AEDF model to include these parameters, with minimal adjustments to the model’s structure. For instance, fundus image data can be incorporated by reshaping the image to a vector and concatenating with other data types as the input to the AEDF model. Furthermore, by modifying the target of the encoding loss function, one can adapt the AEDF model for different data interpretation and analysis purposes. For example, if the encoding loss function in this study was to compute the difference between the encoding and RNFL thickness data during model training, the resulting AE-fused data would be similar in structure to that of the RNFL thickness data while incorporating features associated with perimetric data. In this case, the AE-fused data will be analyzed by standard clinical methods for interpreting RNFL thickness data. Therefore, the unsupervised nature of the AE model and the flexibility in the design of the encoding loss function can collectively make the AEDF model a promising candidate for generalized data integration approach in glaucoma management. 

It should be noted that to accommodate the relatively low-dimensional space of the input data in this study (in contrast to image-based data), we designed the encoder and decoder networks of the AEDF model with a lightweight, simple architecture, i.e., multilayer perceptron with two hidden layers. This approach enhances the robustness and generalizability of the model by avoiding the capture of noise or irrelevant features in the training data, i.e., overfitting, leading to improved performance when applied to unseen data. For a different task with more complex data inputs, a comprehensive investigation of the model architecture and the optimal set of weights for the loss function is imperative. Additionally, in this study, we focused on utilizing the encoder component of the AEDF model for compacted representations of data from VF and OCT tests. As the AE-fused data contains sufficient information from both modalities, the decoder component of the trained AEDF model can be used for simulation purposes, such as generating RNFL thickness profile data based on the corresponding VF measurements. In this case, autoencoder models that excel in generative tasks, such as variation autoencoder [29] and adversarial autoencoder [30], may warrant further investigations.

Constructing the AE fused data in a structure that is similar to VF data provides an intuitive understanding of how OCT data is combined and integrated into perimetric data. The examples in Figure 2 demonstrate that the AEDF model can dynamically combine information from VF and OCT tests in glaucoma patients that are at different stages of the disease. This capacity is particularly important in the context of glaucoma management, as measurements from functional and structural testing modalities may hold distinct clinical significance at different stages. It is typically believed that RNFL thickness measurements are more sensitive to subtle changes in the early stage of glaucoma, while VF measurements have a broader dynamic range that can better support monitoring glaucoma progression in moderate-to-advanced cases [3,4]. For the early-to-moderate glaucoma cases, the RNFL thickness data provides the complementary information to improve the robustness of VF measurements, e.g., to emphasize the depth of VF defects based on corresponding structural damage (Figure 2A) or to remove artifacts in VF measurements (Figure 2B). In advanced glaucoma cases where RNFL thickness data plateaued, the dynamic data integration ability of the AE data fusion model reduces the impact of overly stabilized RNFL thickness data on the AE-fused data. In comparison, the BLR model combines structural and functional progression rates with hard-coded rules that are based only on the uncertainty of the estimates. For eyes with moderate to severe loss (e.g., Figure 2C), the posterior VF progression rate of the BLR model is likely underestimated due to low variability in the plateaued RNFL thickness data. This might explain the lower sensitivity to detect VF progression with data from the BLR model (Figure 3).

This study has several limitations. One of the main limitations is the absence of a reliable and generalized definition of VF progression, especially in early or mild progression. In the study, we coped with this limitation by generating labels based on all longitudinal VF measurements available for each eye, while performance was assessed based on detections made using subsets of the longitudinal data. As VF data are subject to measurements noise, the progression label derived from the entire VF series may be suboptimal and, hence, may affect the performance evaluation. Furthermore, data used for model training and evaluation in this study were sourced from a single glaucoma clinic. As a result, performance evaluation was limited by the number of available longitudinal VF and OCT data, especially for the progressing cases. Testing with data collected from a single clinic with similar clinical management strategies, such as follow-up and treatments, can also introduce bias in the evaluation of the AEDF model. Future evaluations with external datasets containing a greater number of longitudinal data would be essential to comprehensively understanding the generalization of the AEDF model.

## 5. Conclusions

In this study, we developed an autoencoder data fusion model aimed at learning compact encoding (the AE-fused data) from functional VF data and structural OCT data. In the model training, we introduced an encoding loss to ensure that the AE-fused data can be interpreted in a manner similar to the VF data. Comparisons with the clinical standard method to detect VF progression and the Bayesian linear regression model that integrates structure-functional data showed a significant improvement in the specificity of VF progression detection when using AE-fused data. The unique capability of the AE data fusion model to generate interpretable fused data holds the potential to improve its clinical usability. The flexibility of the autoencoder model makes it as a promising candidate for a generalized data integration model to aid in glaucoma management.

## Figures and Tables

**Table 1:** Data Characteristics.

Measurements	Mean (Standard Deviation)	Median (Interquartile Range)
Age (years)	63.7 (11.8)	65.7 (56.4 to 71.8)
Follow-up years	7.7 (1.7)	8.1 (6.8 to 8.8)
Number of visits	9.9 (3.7)	10.0 (7.0 to 13.0)
Initial mean deviation ^1^ (dB)	−3.2 (5.8)	−1.4 (−4.2 to 0.4)
Initial mRNFLT ^2^ (µm)	78.7 (14.4)	78.3 (66.9 to 89.8)
MD slope ^3^ (dB/year)	−0.21 (0.44)	−0.15 (−0.33 to 0.02)
mRNFLT slope ^4^ (µm/year)	−0.24 (0.97)	−0.24 (−0.58 to 0.10)

Note: ^1^ The mean deviation (MD) in the first visual field testing. ^2^ The mean retinal nerve fiber layer thickness (mRNFLT) in the first optical coherence tomograph test. ^3^ The linear regression slope of the longitudinal MD measurements in each eye. ^4^ The linear regression slope of the longitudinal mRNFLT measurements in each eye.

**Table 2:** Performance of visual field (VF) progression detection with different criteria.

Criteria ^1^	Metrics	AE-Fused Data ^2^	Measured Data	BLR Data
<−0.2 dB/year	Specificity	0.67 ± 0.01	0.34 ± 0.01	0.50 ± 0.01
	Sensitivity	0.53 ± 0.01	0.56 ± 0.01	0.51 ± 0.02
	F1 score	0.62 ± 0.01	0.50 ± 0.01	0.52 ± 0.02
<−0.5 dB/year	Specificity	0.70 ± 0.01	0.36 ± 0.01	0.55 ± 0.01
	Sensitivity	0.53 ± 0.03	0.54 ± 0.02	0.35 ± 0.02
	F1 score	0.60 ± 0.01	0.45 ± 0.01	0.48 ± 0.02
<−1.0 dB/year	Specificity	0.70 ± 0.01	0.36 ± 0.02	0.55 ± 0.01
	Sensitivity	0.41 ± 0.07	0.49 ± 0.06	0.27 ± 0.04
	F1 score	0.50 ± 0.03	0.37 ± 0.02	0.44 ± 0.03

Note: ^1^ The criteria for visual field (VF) progression are based on the value of mean deviation linear regression slopes. ^2^ The three data columns show the performance of detecting VF progression with data from the autoencoder data fusion model (AE-fused data), the measured visual field data (Measured data), and the data from the Bayesian linear regression model (BLR data) in the initial 2 years of the follow-up period, respectively. The performance metrics are presented in the form of mean ± standard error of the mean.

## Data Availability

The data used in this study is not publicly available due to ethical restrictions. The code, trained models, and examples are publicly available on GitHub: https://github.com/lcapacitor/glaucoma-vf-oct-data-fusion.

## Appendix A

Bayesian linear regression model:

The Bayesian linear regression (BLR) model is based on the work of Russell et al. [12]. In this study, the mean retinal nerve fiber layer thickness (mRNFLT) data from optical coherence tomography (OCT), representing the structural changes, were utilized to derive the prior distribution of the progression rates. The mean deviation (MD) data from visual field (VF) tests, representing functional changes, were used for the likelihood of the progression rates. Then, the posterior progression rates, i.e., the combination of structural and functional changes, was derived through a weighted average of the structural and functional progression rates. Note that the progression rate was represented by the slope of the linear regression line.

Following Russell et al.’s method [12], before estimating the prior distribution of progression rates, the mRNFLT data (in μm) in our study were first converted into the same scale as MD measurements (in dB). This transformation was achieved by fitting a Passing–Bablok linear regression model [31] to MD and mRNFLT measurements. The resulting linear model based on our data can be expressed as: $MD_{RNFL}=0.241\times mRNFLT-21.310$, where $MD_{RNFL}$ represents the MD value estimated by the corresponding mRNFLT data.

For computational simplicity, we employed the conjugate prior to derive the posterior distribution of the progression rate [32]. Specifically, we assumed that MD progression rates follows Gaussian distribution, denoted as $N(\mu_{1},\;\sigma_{1}^{2})$, where $\mu_{1}$ and $\sigma_{0}^{2}$ are the mean and variance parameters. Here, both $\mu_{1}$ and $\sigma_{1}^{2}$ were derived by fitting an ordinary least square linear regression (OLSLR) model to the measured longitudinal MD data for each eye, where $\mu_{1}$ is the measured slope and $\sigma_{1}^{2}$ is estimated by $MSE/S_{xx}$ from the OLSLR model. Specifically, MSE is the mean squared error (or average squared residual) of the OLSLR model and can be derived from $MSE=\frac{1}{n-2}\sum_{t=1}^{n}{\left(y_{t}-{\hat{y}}_{t}\right)}^{2}$, where $y_{t}$ and ${\hat{y}}_{t}$ represent the measured MD value at time *t* and predicted MD from the OLSLR model at the same time *t*, respectively, and *n* is the total number of MD measurements (visits) in this time series of MD. $S_{xx}$ is the sum of squares of x from the OLSLR model and can be calculated through $S_{xx}=\sum_{t=1}^{n}{\left(x_{t}-\bar{x}\right)}^{2}$, where $x_{t}$ and $\bar{x}$ represent the patient’s age at visit *t* and mean age over the *n* visits for this patient, respectively.

When further assuming that $\sigma_{1}^{2}$ is a constant, the structural progression rates (i.e., progression rates of $MD_{RNFL}$) follows Gaussian distribution, denoted as $N(\mu_{0},\;\sigma_{0}^{2})$, where $\mu_{0}$ and $\sigma_{0}^{2}$ are the mean and variance parameters of the prior distribution. Likewise, $\mu_{0}$ and $\sigma_{0}^{2}$ can be derived by fitting an OLSLR model to the longitudinal $MD_{RNFL}$ data for the same eye, following the same method elaborated above. Therefore, the posterior distribution of progression rate also follows Gaussian distribution, denoted as $N\left(\mu ,\;\sigma^{2}\right)$. The parameter μ of the posterior distribution represents estimated progression rate that combines changes in both structural and functional measurements. It can be analytically derived from a weighted average of the functional progression rate ($\mu_{1}$) and the structural progression rate ($\mu_{0}$), with the weights determined by the variances of these progression rate distributions (Equation (A1)).

(A1) $$\mu =\frac{\sigma_{0}^{2}}{\sigma_{0}^{2}+\sigma_{1}^{2}}\mu_{1}+\frac{\sigma_{1}^{2}}{\sigma_{0}^{2}+\sigma_{1}^{2}}\mu_{0}$$

In other words, whether functional or structural changes, the distribution with the lower variance receives a higher weight in determining the posterior progression rate.
